# A combined experimental and computational analysis of mantATP turnover in skinned muscle fibers

**DOI:** 10.1073/pnas.2502652122

**Published:** 2025-05-15

**Authors:** Mauro Montesel, Cosimo De Napoli, Luisa Schmidt, Elena Germinario, Ulises H. Guzman, Jesper V. Olsen, Lorenzo Marcucci, Leonardo Nogara

**Affiliations:** ^a^Department of Biomedical Sciences, University of Padua, Padua 35131, Italy; ^b^Veneto Institute of Molecular Medicine, Padua 35129, Italy; ^c^Mass Spectrometry for Quantitative Proteomics, Proteomics Program, The Novo Nordisk Foundation Center for Protein Research, Faculty of Health and Medical Sciences, University of Copenhagen, Copenhagen DK-2200, Denmark; ^d^Department of Pharmaceutical and Pharmacological Sciences, University of Padua, Padua 35131, Italy

**Keywords:** myosin, mantATP chasing, ATPase, skeletal muscle, modeling

## Abstract

Myosin is the primary motor protein in skeletal muscle, responsible for adenosine triphosphate (ATP) hydrolysis that drives muscle contraction. In addition to force production, resting myosin consumes ATP in futile cycles at two rates, the slower one being associated with the Super Relaxed State (SRX), in contrast to the less inhibited Disordered Relaxed State (DRX). The SRX is typically measured using the mantATP chasing technique, where the decay of a fluorescent ATP analogue is fitted using a multiexponential function. Recently, significant concerns have been raised regarding the use of this technique, particularly when applied to soluble myosin preparations. While skinned fibers offer the advantage of preserving the native thick filament structure and myosin cooperativity, limited diffusion and nonspecific mantATP binding pose challenges. In this study, we combine experimental data and in-silico modeling to dissect the contributions of different components in the mantATP chasing signal. We analyze control skinned fibers and fibers subjected to myosin extraction. Our analysis shows that the nonspecific component partially overlaps with the DRX timescale. In contrast, the slow component linked to myosin SRX nucleotide release is characterized by a time constant that significantly differs from those of the nonspecific signal and DRX, enabling its reliable estimation using this technique. Our findings indicate that evaluating nonspecific mantATP components is necessary to obtain a reliable estimation of both SRX and DRX. We validated our analysis by comparing populations and time constants obtained from chasing with mantATP to mantATPase rates in control conditions and upon piperine-induced SRX destabilization.

## Significance

Myosin resting states play a critical role in regulating muscle contractility and basal metabolism. The mantATP chasing technique has been widely used to assess the nucleotide turnover of resting myosin associated with the Super Relaxed State (SRX). However, recent studies have raised questions about the reliability of results obtained using this technique across various models. Given the growing interest in understanding myosin resting energy consumption, we focused on skinned fibers to better characterize the limitations of this method and to develop a more reliable analytical approach for estimating SRX. Accurate interpretation of myosin resting states is essential for a range of research fields, from drug development to clinical evaluation, where insights into muscle function and energy dynamics are critical.

Myosin is the skeletal muscle motor protein responsible for converting the energy of ATP into mechanical work. During muscle contraction myosin ATPase activity is promoted by its binding to actin, while during resting the nucleotide turnover is extremely limited. At rest, two biochemical states with different ATP turnover are populated by myosins: the slower was named “Super Relaxed State” (SRX), while its disordered counterpart was named “Disordered Relaxed State” (DRX) (1). At its definition time, the biochemical state and myosin orientation seemed to be tightly connected, with the Interacting Heads Motif (IHM) being the structural identity of the inhibited complex (2). IHM structural conformation slightly varies between species and myosin isoforms (3), but can be described as the folding back of two myosin heads onto the thick filament axis through a head–head interaction. This conformation is asymmetric, leading to a more internal blocked head and a more accessible free head (4, 5). The structural organization of IHM has been confirmed in several myosin preparations with different techniques (2, 5, 6), while DRX myosin conformation remains more elusive. Traditionally, the “disordered” adjective of DRX was due to the structural correlation between biochemical states, and as such, it represented the opposite to the ordered IHM/SRX. The association between myosin structure and biochemical state has lately become more loosely defined (6–8), but it is possible to hypothesize that the structural features do play a role in supporting possible different biochemical configurations (9). These biochemical states can be identified through the mantATP chasing experiment. In a classical chasing experiment on skinned fibers, a single fiber is incubated in a buffer containing mantATP (a fluorescent ATP analogue) so that the nucleotide saturates all myosins. Then, the buffer is quickly replaced with a fresh one containing standard ATP. The mantATP is hydrolyzed to mantADP and released according to the turnover rate of the myosin head it was bound to. The decrease in fluorescence intensity is fitted to obtain myosin populations and time constants.

Since its use for the determination of myosin SRX state itself (1), the mantATP chasing has been widely used as a gold standard technique to quantify both the relative populations and the hydrolyzation time constant of the SRX and DRX states in different kind of preparations, ranging from the muscle fiber to the single motor domain. However, recent experimental and theoretical arguments urged caution in using the fluorescence decay as the sole source of information about the SRX and recommended a detailed control to avoid possible limits in the fitting procedure (10), especially in purified proteins. The main “paradox” is due to the fact that SRX and DRX myosin are generally considered in thermodynamic equilibrium. However, if this is the case, the fluorescent decay should be described by a single exponential. Besides these theoretical considerations, the authors showed, contrary to previous reports, a single exponential decay in several purified myosin preparations (10). Yet, a biphasic decay occurs in mantATP assay when myosin heads are assembled in native thick filaments, possibly due to a cooperativity among motors. While the skinned fiber model retains all the cooperative and structural features of physiological myofilaments, it suffers from other limits. In this work, we showed that slow mantATP diffusion and nonspecific binding interfere with SRX/DRX populations, and that an independent evaluation of those components is necessary to obtain a reliable estimation of both populations. To validate our findings, we solved the paradox of estimating ATPase rates based on chasing mantATP parameters.

Notably, the physiological mechanism involving myosin resting states pioneered by Cooke et al. recently gained a large interest in the cardiac field (11, 12), while in skeletal muscle it is still in its early stages (13). In this respect, skinned fibers represent an accessible model that can be applied to human patients for clinical characterization, as well as for the development of new molecules of therapeutic interest.

## Results

### Cooperativity in Thermodynamically Equilibrated Myosin Populations Generates a mantATP Biphasic Decay.

We used a Monte-Carlo approach to simulate how different intra- and intermolecular cooperativity hypotheses affected the hydrolyzation of the mantATP in a sarcomere. In all models the rates of ATPase are 0.05 s^−1^ for the DRX motors and 0.005 s^−1^ for the SRX motors.

**Fig. 1.**
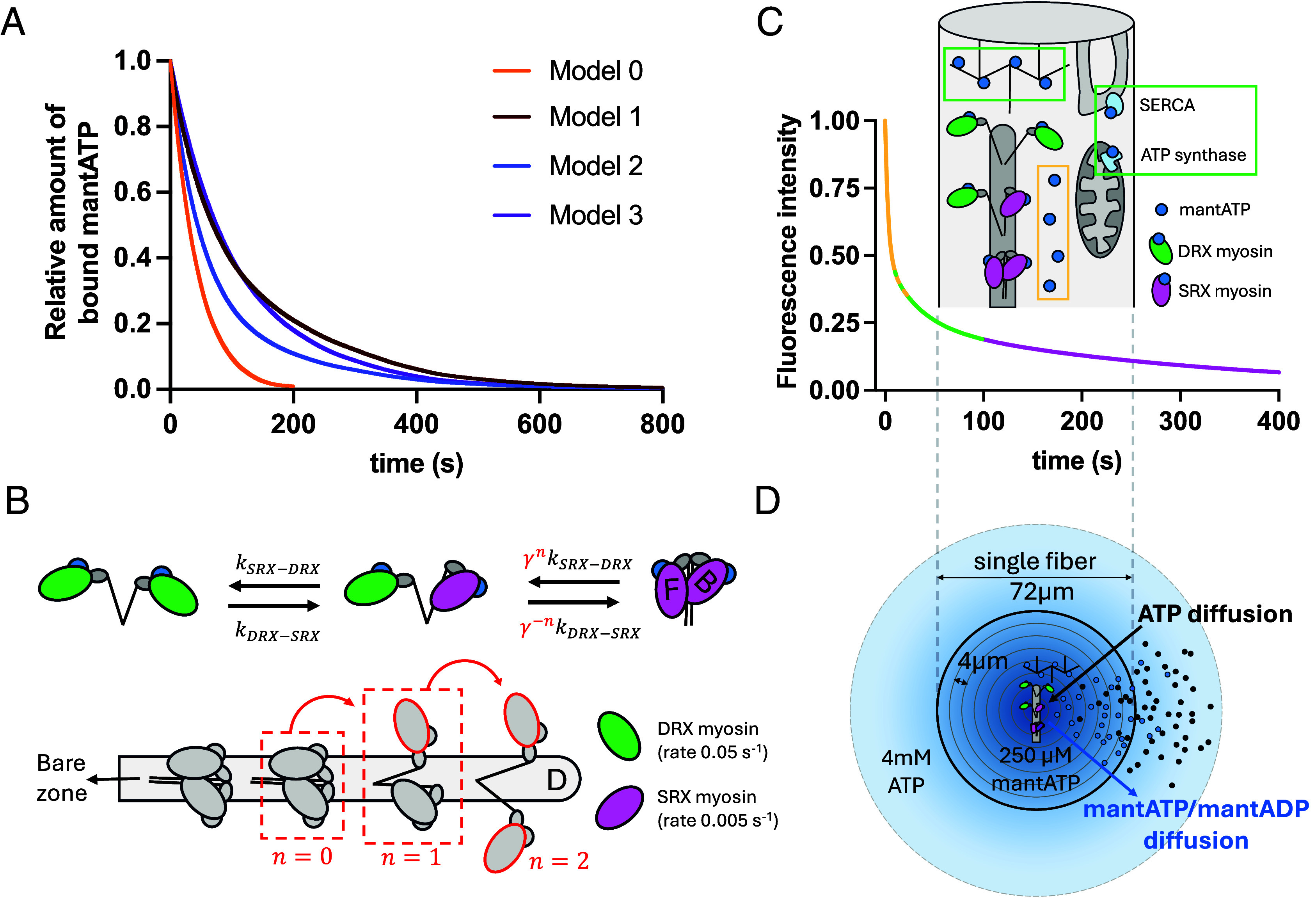
Experimental scheme and model. (*A*) Simulated amount of bounded mantATP in a single sarcomere under different hypotheses for the cooperativity between myosin motors. Model 0: noncooperative thermodynamically equilibrated motors. Model 1: two populations of motors with diverse thermodynamic equilibrium. Model 2: only intradimer cooperativity with a forbidden state (free head in SRX with blocked head in DRX; see below). Model 3: full model with both intradimer and intermolecular cooperativity as detailed in panel *B*. (*B*) Kinetic diagram relating the transition rates to the motors state in each dimer (*Upper* panel, intradimer cooperativity) and along the filament (*Lower* panel, intermolecular cooperativity). Intradimer cooperativity is simulated preventing a dimer configuration with a free motor in the SRX state (SRX states are indicated in purple and have ATPase rate of 0.005 s^−1^) and a blocked motor in DRX state (DRX states are indicated in green and have ATPase rate of 0.05 s^−1^). (*C*) Schematic representation of a fluorescence decay trace: fast diffusing free mantATP (yellow), a combination of mantATP binding nonspecifically to structural elements and nonmyosin ATPases, as well as myosin in DRX (green), myosin in SRX (purple). Trace is built using the following parameters P_NSP_ = 0.5; T_NSP_ = 3 s; P_DRX_ = P_SRX_ = 0.25; T_DRX_ = 20 s; T_SRX_ = 200 s). (*D*) Schematic representation of the geometry applied to the model of mantATP release and diffusion. Fiber (black circle) is divided into nine concentric cylinders. The scheme has been prepared using Microsoft PowerPoint licensed to LN and LM, University of Padova (Italy).

In the first model, each myosin head was stochastically fluctuating between SRX and DRX states with no cooperativity between heads. In the model, this situation is simulated imposing the same *k*_SRX-DRX_ and *k*_DRX-SRX_ rates for all motors (see *SI Appendix*, Fig. S1, Model 0). Coherently with theoretical arguments (10), this situation simulated a fluorescence decay that could be fitted by a single exponential, with a time constant that was an intermediate between the two values imposed in the model (Model 0 in Fig. 1*A* and *SI Appendix*, Table S1). The simplest way to obtain two exponential decays was to impose a nonequilibrium model (10), where each head stayed in only one biochemical state in relaxed condition, without interconversion between SRX and DRX (*k*_SRX-DRX_ = *k*_DRX-SRX_ = 0). This simple nonequilibrium model led to a clear biphasic decay of the simulated fluorescence in relaxed sarcomere, and the two exponential fitting was able to predict the parameters imposed in the model (Model 0A in *SI Appendix*, Fig. S1 and Table S1).

However, a model in which the SRX and DRX populations were completely out of equilibrium is not strictly needed to generate a two-exponential decay. It is sufficient to include (at least) two “sets” of motors with different *k*_SRX-DRX_ and *k*_DRX-SRX_ rates, i.e., with different stability of the two states. This situation was simulated in model 1 and could be associated with the presence of a stabilizing protein, like the Myosin binding Protein C (MyBP-C), or with different *k*_SRX-DRX_ and *k*_DRX-SRX_ rates in the blocked and free heads, even in absence of intramolecular or interdimer cooperativity. In each set, the myosin motors hydrolyzed the mantATP generating a single-exponential decay but characterized by a time constant related to the stability of the DRX and SRX states in that set of motors, as seen in model 0. At the fiber level, the two sets of motors generated a two-exponential decay (Fig. 1*A* and *SI Appendix*, Fig. S1 and Table S1). However, in these cases, a change in the SRX-DRX stability within the two sets of motors could modify the time constant estimated in the double-exponential fit, but not the relative amplitudes of the populations, which are defined by the number of motors within each set of motors.

Since different external perturbations have been shown to affect the relative amplitudes of the exponential fitting in mantATP chasing experiments, in the next models we introduced cooperativity between heads. Several hypotheses were possible, both with and without a direct link between structural and biochemical states, and our method could not distinguish between them. As a simple approach, we imposed the same *k*_SRX-DRX_ and *k*_DRX-SRX_ rates for both blocked and free motors, and we introduced the structural constraint that an SRX blocked motor cannot switch to DRX unless its free motor is already in the DRX state. Similarly, a free motor in the DRX state cannot switch to SRX state unless its blocked motor is already in the SRX state. This kind of intradimer cooperativity was enough to create a perturbation in the relative stability between SRX and DRX states of the free and blocked motors, creating a multiexponential fluorescence decay (Model 2 in Fig. 1*A* and *SI Appendix*, Fig. S1 and Table S1). Then, we also introduced intermolecular cooperativity using the structural insights observed in cryo-electron microscopy studies (14, 15). The free head SRX state was favored through a factor *γ* (Fig. 1*B*) when the closest dimer toward the M-line had one or two heads in the SRX state, following a cooperativity model previously proposed for the active motors (16). This approach created a multiexponential decay (Model 3 in Fig. 1*A* and *SI Appendix*, Fig. S1 and Table S1). The relative populations estimated by the exponential fitting of the decay can be affected by cooperativity perturbations, because the decay was generated by a continuously increasing SRX state stability along the thick filament due to the intermolecular cooperativity. In other words, Model 3 defined a continuum of differently stable SRX states coexisting on a thick filament.

The previous models analyze the mantATP hydrolysis in a single sarcomere, but at the fiber level, released mantADP must also diffuse away and there could be nonspecific binding sites possibly slowing the fluorescence decay. To understand how these components influence the reliability of the estimation of the SRX and DRX populations, we used a reaction–diffusion model. A scheme of our analytical approach is reported in Fig. 1 *C* and *D* in which the fluorescence decaying signal obtained from an ideal mantATP chasing experiment of a skinned fiber is divided into different components. The initial fast decay is associated with diffusion of free nucleotide (yellow), the second phase is associated with the nucleotide that is bound to both nonspecific targets (structural component or other ATPases) and fast cycling myosin in DRX state (green). The last component is associated with myosin heads in the SRX state with a slow nucleotide release (violet). To simulate the whole decay, reported later, we fed the model with experimentally constrained parameters, as described in the next section.

### Ghosted Fibers Revealed a Nonspecific mantATP Fluorescent Emission of About 60%.

A known limitation of mantATP chasing experiment in skinned fibers is the relatively large amount of nonspecific signal. This signal can be originated by soluble mantATP, or to that bound to nonmyosinic targets. To study the contribution of myosin and nonmyosin fluorescent nucleotide emission, we analyzed the signal of control fibers and in fibers in which myosin was extracted (ghosted) using confocal microscopy in 250 µM mantATP. This situation resembled that established at the time zero in the fluorescence decay, when the fiber is surrounded by fresh medium, but it still has inside 250 µM mantATP in solution. This point was particularly important since its net fluorescent emission was used to normalize all the following data points.

**Fig. 2.**
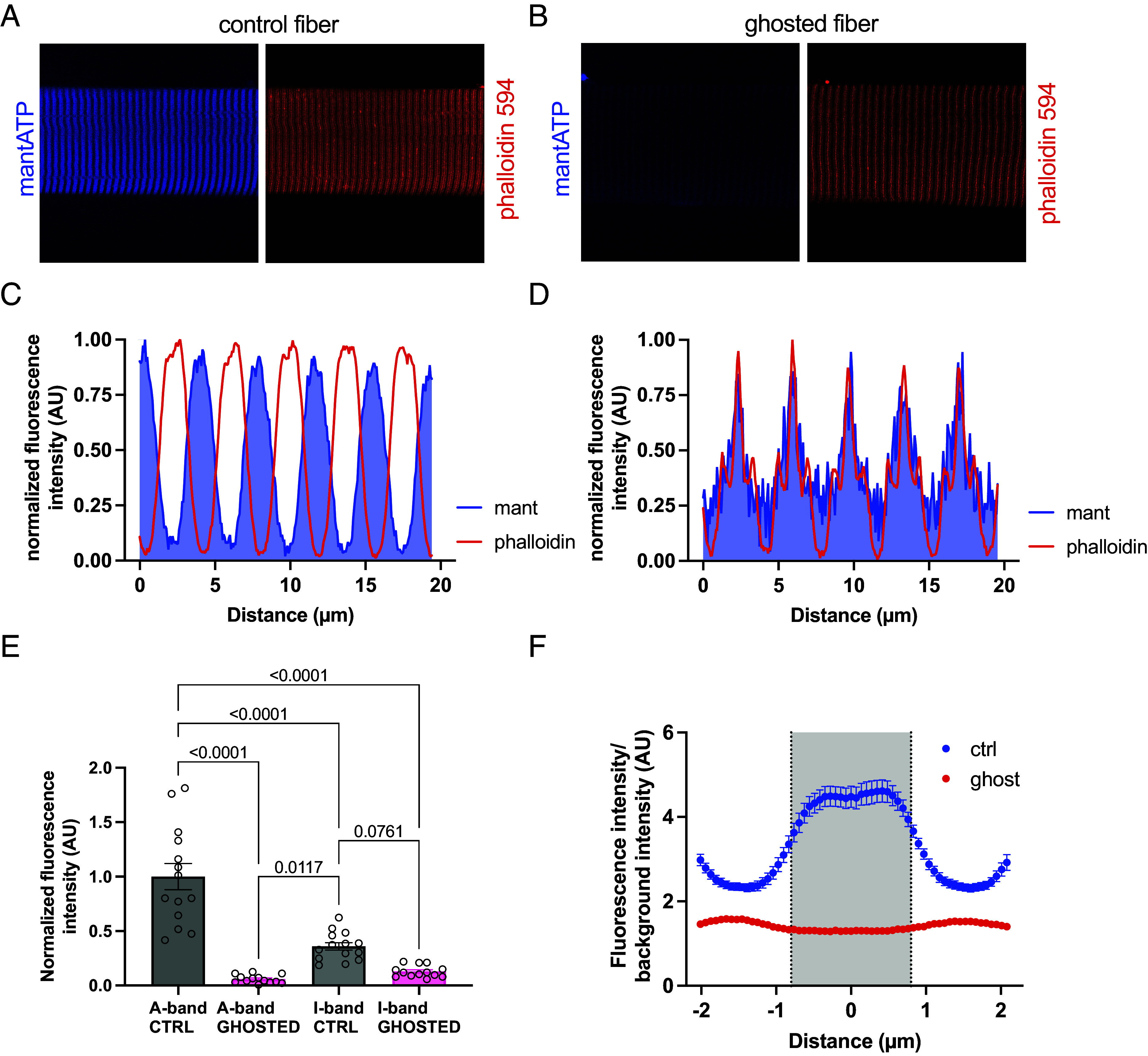
Myosin extraction on ghosted fiber and mantATP fluorescence emission patterns. (*A*) Control skinned fiber and (*B*) ghosted fibers incubated with 250 µM mantATP (blue is mantATP, red is Phalloidin, magnification 63×). (*C*) Plot profiles of a control fiber showing the alternance of A-bands (mantATP, blue) and I-bands (phalloidin, red) of five sequential sarcomeres. (*D*) Plot profiles of a ghosted fiber showing that the remaining mantATP signal left is located within the I-band (mantATP, blue, and phalloidin, red). (*E*) Normalized fluorescence intensities of A-band and I-band of control (CTRL) and ghosted (GHOSTED) fibers in 250 µM mantATP (n = 14 control, n = 13 ghosted fibers, mean ± SEM). (*F*) Intensity profile obtained from averaged single sarcomeres in control and ghosted fibers, normalized for background intensity, A-band is highlighted by the gray area (n = 14, mean ± SEM).

**Fig. 3.**
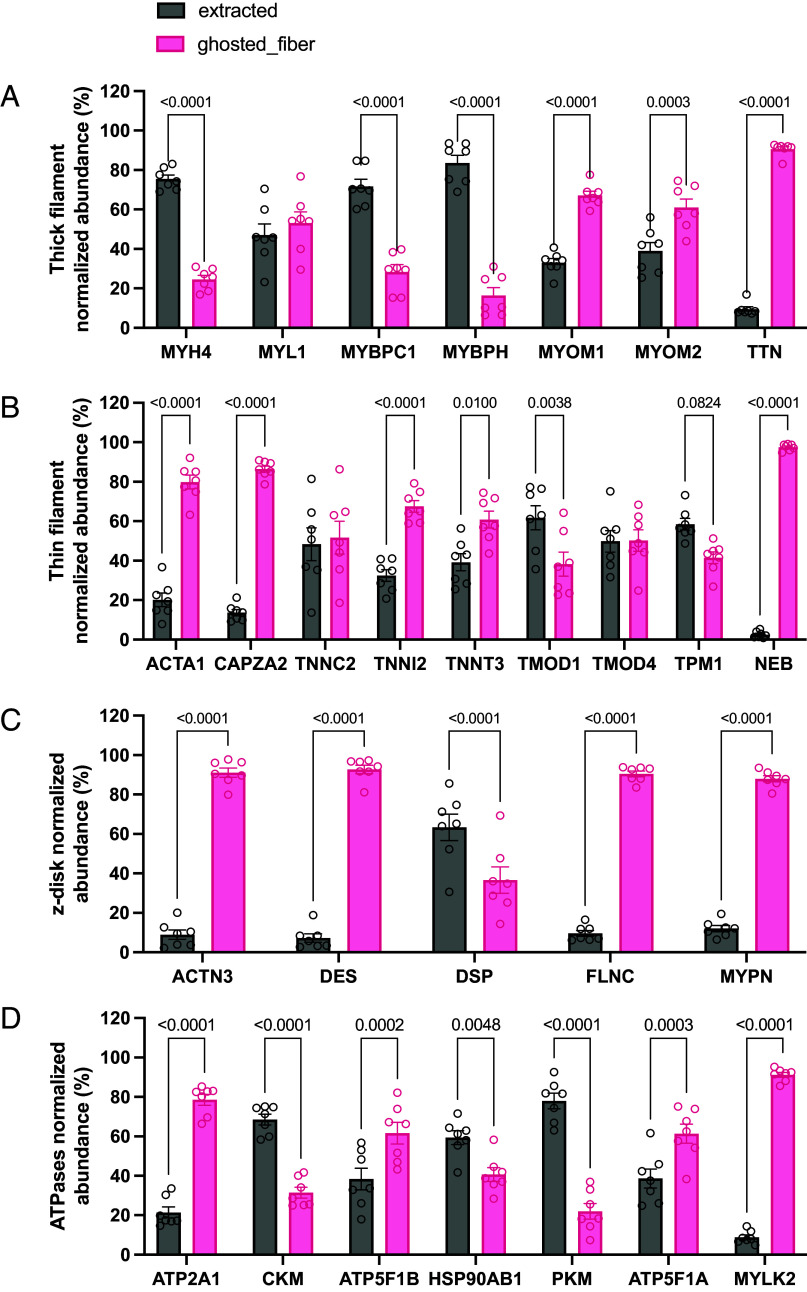
Mass spectrometry analysis of extracted protein and ghosted fibers. Normalized protein amounts in extract and in the ghosted fiber, proteins are divided into thick filament (*A*), thin filament (*B*), z-disk (*C*), and ATPases (*D*). Individual intensities are normalized for the sum of extract and corresponding ghosted fiber intensity, statistical analysis is ordinary two-way ANOVA and Šidák multiple comparison test (n = 7, mean ± SEM).

To explore the behavior of mantATP diffusion in a fiber like environment but without its primary binding site, we prepared “ghosted” fiber in which myosin was extracted using 0.5 M potassium chloride and 0.01 M sodium pyrophosphate in relaxing conditions (17, 18). The extraction efficiency was estimated by silver staining as 5.07 ± 0.76% of myosin left within the fiber (*SI Appendix*, Fig. S2 *A* and *B*). In control fibers, the majority of mantATP emission signal was located on the A-band (Fig. 2 *A* and *C*). Despite being significantly diminished (Fig. 2*E*), ghosted fibers showed a different distribution of mantATP fluorescence emission, which was located on the I-band as highlighted by the phalloidin staining (Fig. 2 *B* and *D*). The ghosting procedure decreased the mantATP fluorescence emission of the A-band to 6.00 ± 0.04% (mean ± SD), a value that is in line with the myosin quantification obtained by silver staining (*SI Appendix*, Fig. S2 *A* and *B*). In Fig. 2*F*, the “sombrero” graph shows the fluorescent intensity ratios of averaged sarcomeres in control and ghosted fibers normalized for the background intensity. The averaged values for control fibers highlighted a small dent on the H-zone which became visible on individual acquisitions based on fiber sample orientation and alignment. The emission of mantATP bound to myosin was not confined to the thick filament, as it exceeded the 1.6 µm in length (19). We hypothesized that a certain amount of scattering from the A-band contributed to the increase in signal, as with the extraction of myosin whose intensity was strongly diminished. In Fig. 2, sarcomere length was about 3 µm since the fibers were slightly stretched to help separating signals at the confocal microscope. To better understand our ghosted model, we analyzed the protein content of the extract and that of the corresponding ghosted fibers by mass spectroscopy (Fig. 3 and *SI Appendix*, Fig. S3). In this analysis, we obtained a myosin extraction of about 75%, but we also identified many proteins that were removed by the high salt buffer. Some structural proteins within the thick filament such as myosin light chain, myosin binding protein C and myosin binding protein H were extracted to values comparable to that of myosin. The giant proteins of the thick filament titin and of the thin filament nebulin were very solidly secured to the cytoskeleton, measuring only minimal amounts in the extraction buffer (Fig. 3 *A* and *B*). Other small structural proteins belonging to the thin filament showed low extraction (ACTA1 and CAPZA2), while more unstable proteins such as troponins (TNNC, TNNI, and TNNT) and tropomyosin were removed to higher extent (Fig. 3*B*). The loss of these proteins, and possible nonspecific mantATP interactions with them, might explain the decrease in I-band fluorescent emission reported in Fig. 2*F*. Structural protein associated with the Z-disk seemed to be resistant to the ghosting protocol (Fig. 3*C*). Considering the stability of the Z-disk proteins, and the scattering emission of mantATP mentioned above, we decided to normalize the intensity values of the sombrero graph (Fig. 2*F*) for that of the Z-line in the confocal pictures reported in Fig. 2. In normalized data, the ratio intensities of averaged sarcomeres in control and ghosted fibers revealed a nonspecific signal estimated to be 60% in saturating mantATP concentrations. Most of this nonspecific signal derived from the large amount of free nucleotide in the buffer permeating the fiber, while a certain amount could be due to mantATP bound to structural elements and other ATPases present in skinned fiber samples. In fact, the emission of mantATP increases when bound to myosin (1, 20), and this result was confirmed by our data. The A-band intensity of control fibers, subtracting that of ghosted fibers and adjusted for myosin and free mantATP concentrations (respectively 105 µM and 250 µM), revealed an increased fluorescence emission of about 10-fold, which was in line with what reported in literature (1, 20). This value was used to model experimental data as explained below. Related to this, it is possible that the increased mantATP emission also occurred when the fluorescent nucleotide was bound to other ATPases. Our proteomic analysis revealed that the most present ATPase in skinned fiber after myosin is the Sarco-Endoplasmic Reticulum Calcium ATPase (SERCA, gene name ATP2A1) which was about two orders of magnitude less abundant than the motor. Despite being measured, other ATPases reported in Fig. 3*D* were progressively less abundant than SERCA, and nonrelevant in the context of mantATP chasing experiment. To exclude SERCA contribution to mantATP chasing and ATPase turnover, the inhibitor thapsigargin was added to the buffer (21). The implication of mantATP possibly binding to other proteins besides myosin is discussed in the next paragraph.

### mantATP Diffusion Kinetics Overlaps with That of Disordered Relaxed Myosin.

**Fig. 4.**
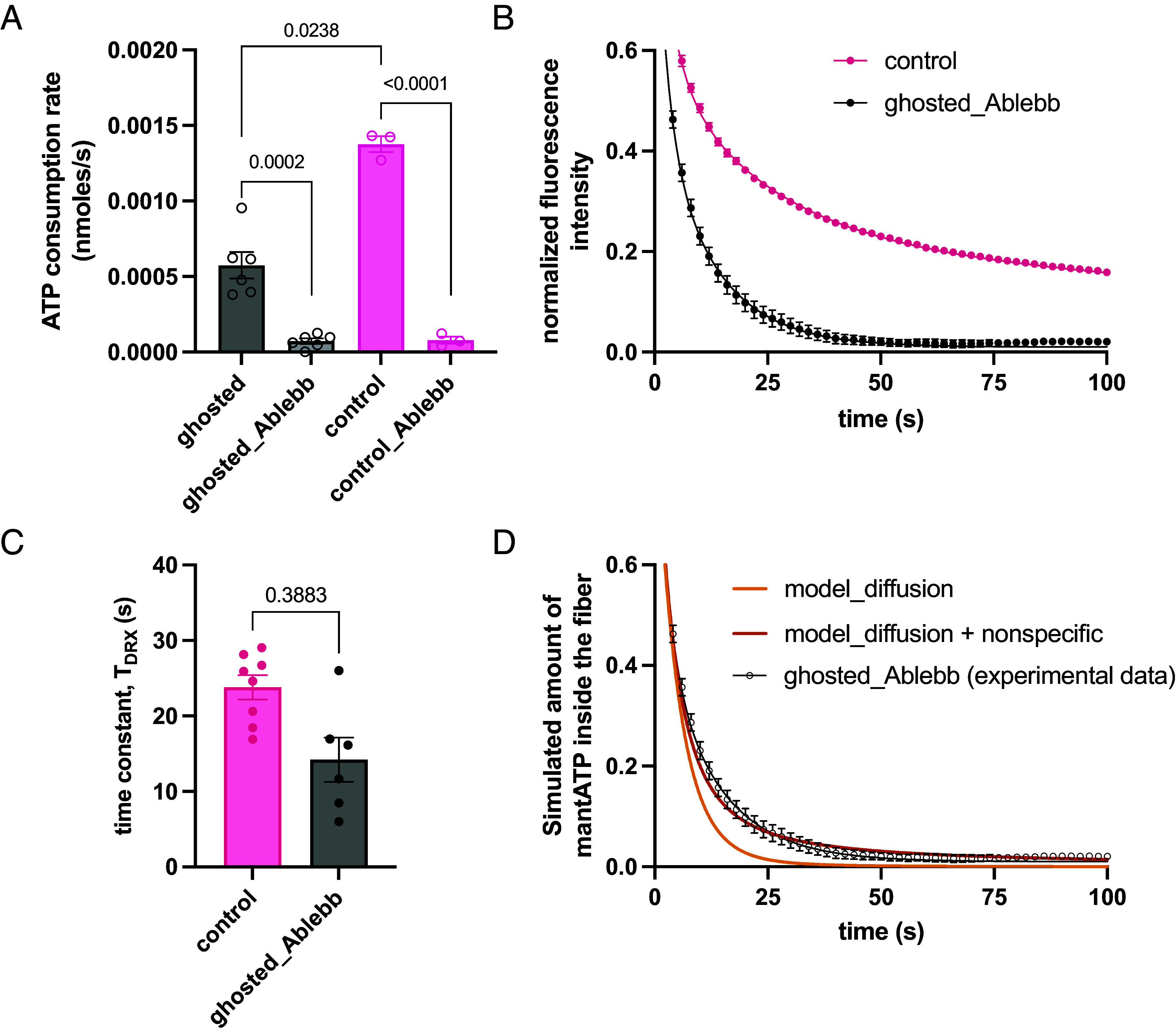
Kinetics of the NSP in mantATP chasing of skinned muscle fibers. (*A*) ATP consumption rates of control and ghosted fibers, with and without 20 µM para-aminoblebbistatin (Ablebb) (n = 3 to 6, mean ± SEM). (*B*) Chasing traces of control fibers and ghosted fibers incubated with 20 µM para-aminoblebbistatin (Ablebb) (n = 6 to 8, mean ± SEM). (*C*) values of time constant T_DRX_ related to curves in panel *B*. (*D*) Simulations of a mantATP chasing experiment on ghosted fibers without the nonspecific buffer (light orange) and with the nonspecific buffer (orange), compared to the experimental data (black curve). The presence of the buffer improves the fitting at higher times (fitting parameters k_ON_ = 10^7^ M^−1^ s^−1^ k_A_ = 5.6 10^4^ M^−1^).

The small amount of myosin retained in ghosted fibers was not able to produce any tension in saturated calcium concentrations, but its resting ATPase activity was surprisingly high. In Fig. 4*A*, we showed the ATPase rate of ghosted and control fibers. We hypothesized that the remaining myosin heads were not subjected to thick filament cooperativity, and thus, no longer inhibited in their resting nucleotide turnover. To understand the contribution of mantATP diffusion in ghosted fibers, we incubated those with the myosin inhibitor para-aminoblebbistatin (22) and performed the chasing experiment (Fig. 4*B*). To compare the fluorescence decay in ghost fibers with para-aminoblebbistatin with that of the control fibers, we first observed that the former did not follow a single exponential decay. Actually, even if all the mantATP nonspecifically bound to myosin would simply diffuse out of the fiber, the analytical solution for the decay would be a summation of exponentials (23). So, we compared the time constant of the second exponential residue of the ghosted decay (black) with the analogue of control fibers (magenta) fitted with a conventional triple-exponential function (Fig. 4*C* and *SI Appendix*, Fig. S4). These results indicated that the time constant T_DRX_ did not significantly differ in the two conditions (Fig. 4*C*), demonstrating that the nonspecific component (NSP) was slow enough to partially overlap with the disordered myosin population. Moreover, the experimental decay cannot even be fitted by a purely diffusing mantATP model as shown by the trace “model_diffusion” in Fig. 4*D*, where a single diffusion coefficient D resulted in a faster than observed decay at higher times. Then, we imposed a nonspecific buffer for mantATP and we used the experimental trace to define its characterizing parameters (Fig. 4*D*, “model_diffusion + nonspecific” trace), used in the next section.

### Three Exponential Fitting Combined with the Estimation of Nonspecific mantATP Better Represents Chasing Data over Other Approaches.

The fluorescence decay of a mantATP chasing assay is usually interpreted using multiexponential decay functions, through the hidden assumption that the two main populations are thermodynamically independent. This assumption allowed us to avoid the Monte-Carlo approach and to use a numerically treatable reaction–diffusion model of a whole fiber to estimate the errors introduced by the three fitting techniques used in literature (*Material and Methods*).

**Fig. 5.**
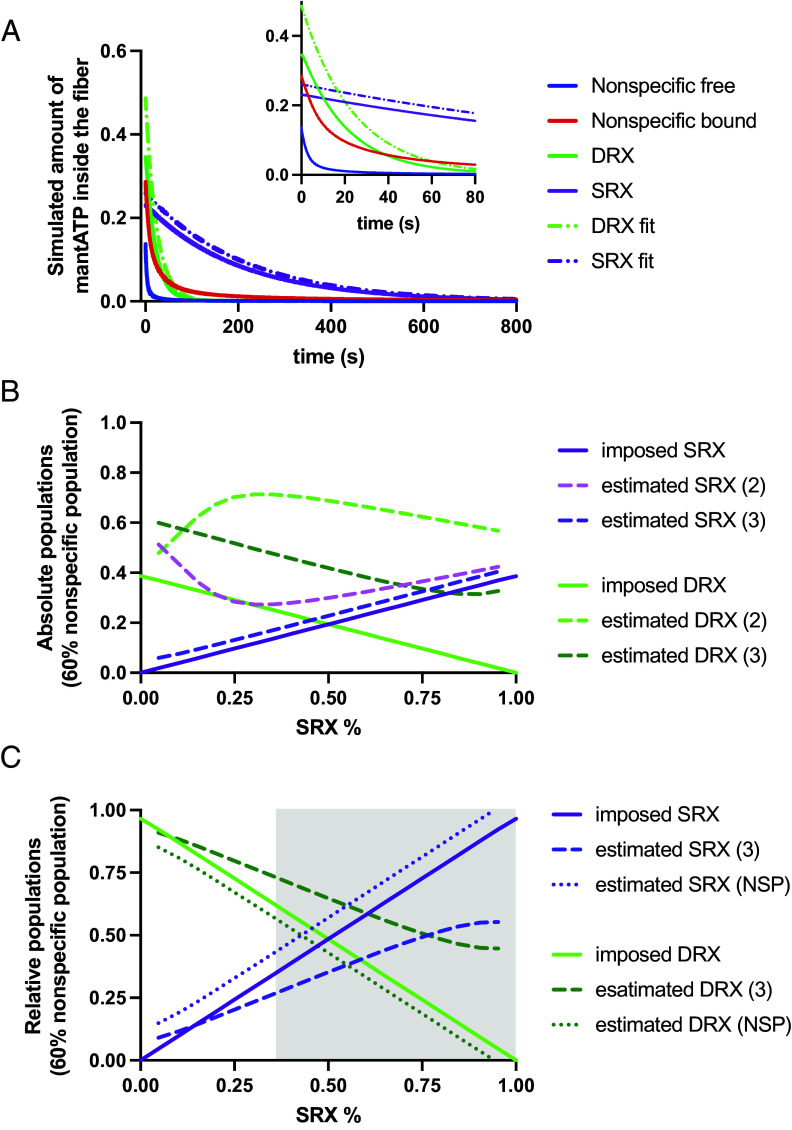
Simulated decay of mantATP concentration. (*A*) Example of the simulated decays of the four components of mantATP inside the fibers: free, nonspecifically bound, bound to DRX, and bound to SRX (continuous lines, blue, red, green, and purple, respectively). Dashed-dot lines represent the estimated decays of SRX and DRX from a three exponential fitting (same colors). (*B*) Absolute SRX-DRX populations imposed on the model (straight lines, purple, and green, respectively) and their estimation through a two (light colors dashed lines) or a three (dark colors dashed lines) exponential fitting at imposed NSP = 60%. (*C*) Relative populations [SRX/(SRX+DRX) and DRX/(SRX+DRX)] and estimated parameters without (dashed lines) and with (dotted lines) the independent information about NSP [estimated DRX = 1−P_SRX_/(1−NSP)]. The gray area located at approximately 37% in the SRX population axis indicates the area in which NSP correction performs better than three exponential fitting to estimate populations.

We imposed in the model the three populations of disordered motors, super-relaxed motors and nonspecific (DRX, SRX, and NSP) and simulated the normalized fluorescence decay. Then, we compared it to the estimated parameters obtained by the fitting procedures (P_DRX_, P_SRX_, and P_NSP_ when present) as shown in Fig. 5*A*. First, we imposed NSP, the total nonspecific signal, equal to 60%, as observed from the confocal analysis, and we varied the relative SRX population (SRX/(SRX+DRX), or SRX/0.4 in this case) from 5 to 95% (*SI Appendix*, Fig. S5).

In absolute terms (Fig. 5*B*), both P_SRX_ and P_DRX_ overestimated the imposed SRX and DRX, being the three exponential estimations (method 2) better than the two exponential ones (method 1). The absolute error was smaller for SRX than for DRX because of the higher time constant of the former, further from the nonspecific time constant. In relative terms (Fig. 5*C*), more useful from a clinical point of view, the estimation of the SRX and DRX populations using the three exponential fitting (method 2) had an error that increased with the imposed SRX population. Instead, assuming that NSP is known from independent experimental data (method 3 of the fitting procedures in material and methods), produced smaller errors at every SRX population (Fig. 5*C*). Our simulations indicate that at a SRX population of 37% or higher, method 3 performs better than method 2. With method 3, DRX was slightly underestimated to compensate for the overestimation of SRX. Globally, the analysis showed that the third approach is more reliable, while others, especially the two-exponential approach, may end in nonreliable results at higher SRX. The estimated time constants were always better in the three exponential approaches (the same for methods 2 and 3).

We also imposed a relative SRX = 50% and we varied the NPS from 20 to 80% (*SI Appendix*, Table S3). This analysis also confirmed that the three exponential fitting was always better than the two exponential fitting in the estimation of both populations and time constants. The third approach was again able to limit the error up to higher values of NSP, reaching a reliable result even at NSP = 60%, the value observed in our confocal analysis.

### Solving a Paradox: mantATP Chasing Parameters Are Representative of Resting ATP Consumption Rates.

**Fig. 6.**
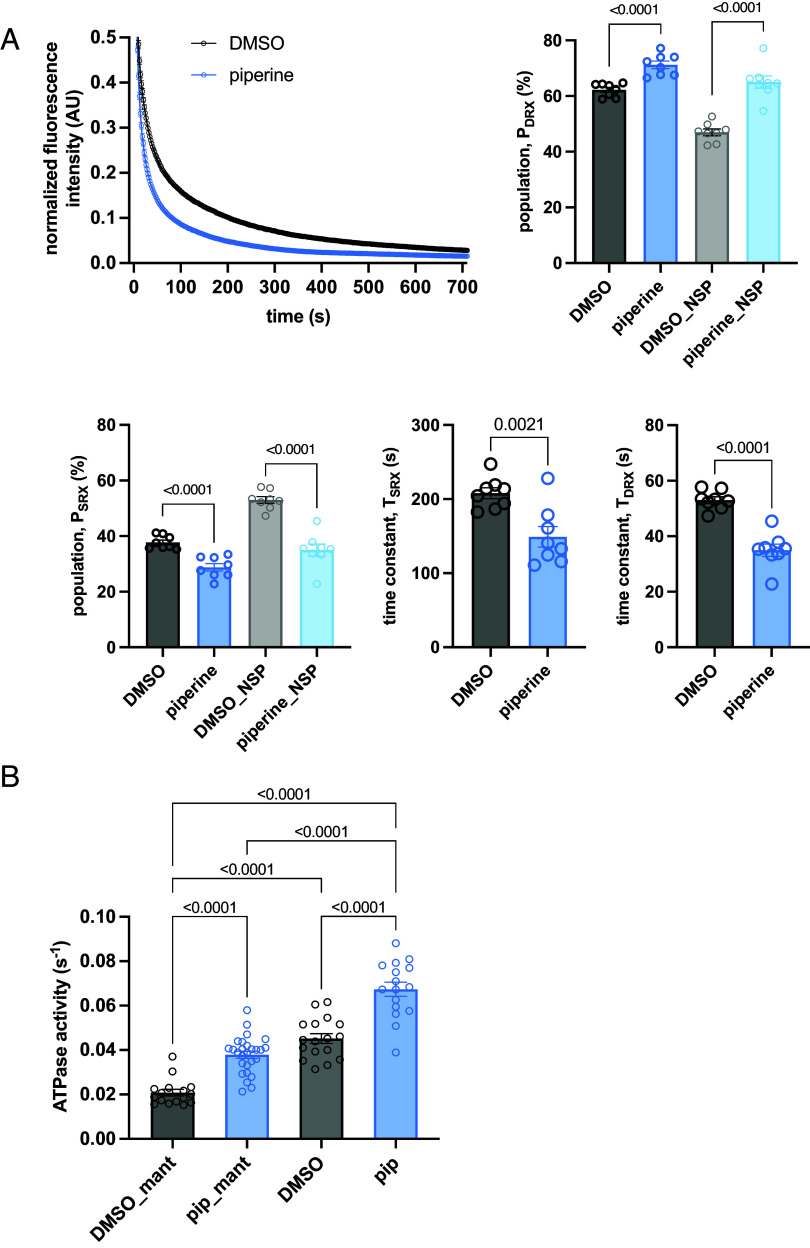
mantATP chasing and NADH coupled ATPase-mantATPase of control and piperine incubated fibers. (*A*) Exponential decay traces of chasing on skinned fibers incubated with DMSO (control) and 50 µM piperine. Histograms are related to estimation of P_DRX_ and P_SRX_ populations by using the triple exponential fitting (darker bars) and correcting the values for the NSP (lighter bars), and histograms related to time constants T_DRX_ and T_SRX_ (n = 8, mean ± SEM). (*B*) Resting skinned fibers ATPase is measured by NADH double-reaction assay on control skinned fibers and incubated with 50 µM piperine using mantATP and normal ATP. Data are analyzed using an identify outliers ROUT method Q = 0.5%, Kolmogorov–Smirnov normality test, ordinary one-way ANOVA, and Tukey’s multiple comparison test (n = 16 to 27, mean ± SEM).

Properly performed, the mantATP chasing assay delivers myosin populations and nucleotide turnover times that, combined, must be aligned with the steady state ATPase rate. To verify this, we compared mantATP chasing results with ATPase assay data. In addition to the control condition [dimethyl sulfoxide (DMSO)], we considered a perturbed situation in which myosin SRX is destabilized by piperine (24). Experimental chasing data of skinned fibers in DMSO and 50 µM piperine were fitted using the standard triple exponential (method 2) or by correcting for the NSP (method 3) obtaining different P_DRX_ and P_SRX_ (Table 1). These values were used to estimate the contribution of each population to the total mantATP consumption rates. In control fibers incubated with DMSO, method 2 led to a calculated mantATPase rate overestimated by 28.5% (estimated rate of 0.0290 ± 0.0033 s^−1^) compared to the measured mantATP consumption rate (0.0208 ± 0.006 s^−1^). The same experimental data interpreted using model 3 limits the error to +8.8% (estimated rate 0.0228 ± 0.0033 s^−1^). Piperine induced a shift in myosin population toward a more DRX state, also significantly reducing time constants of both populations (Fig. 6*A*). As anticipated in Fig. 5*C*, this condition of enriched fast cycling myosin heads improves the reliability of method 2, limiting the error to +7.6% (estimated rate of 0.0410 ± 0.0126 s^−1^ compared to the measured 0.0379 ± 0.0083 s^−1^). The application of method 3 still resulted in the best agreement between estimated and observed values, offsetting the result by only −1.6% (estimated rate of 0.0373 ± 0.0027 s^−1^). The rate of mantATP turnover in DMSO was 2.17 times slower than that of ATP possibly because of the increased affinity for myosin of the fluorescent nucleotide (25). In the presence of piperine, the ATPase/mantATPase ratio showed a reduction to 1.78, suggesting that the ATP analogue might affect myosin populations unevenly (Fig. 6*B*).

**Table 1.** Fitting parameters of experimental mantATP chasing data of fibers in 50 µM piperine and DMSO (control)

	control (DMSO)		50 µM piperine	
	Three exp fitting (method 2)	Three exp fitting + NSP (method 3)	Three exp fitting (method 2)	Three exp fitting + NSP (method 3)
P_DRX_ (%)	62.3 ± 2.4	47.0 ± 3.4	71.2 ± 3.8%	65.0 ± 6.2
T_DRX_ (s)	23.8 ± 4.6		20.1 ± 7.3	
P_SRX_ (%)	37.7 ± 2.4	53.0 ± 3.4	28.8 ± 3.8	35.0 ± 6.2
T_SRX_ (s)	207.8 ± 20.8		149.0 ± 39.2	
DRX rate (1/T_DRX_)	0.0435 ± 0.0092		0.0546 ± 0.0269	
SRX rate (1/T_SRX_)	0.0048 ± 0.0004		0.0070 ± 0.0015	
DRX mantATPase contribution (1/T_DRX_)*P_DRX_	0.0272 ± 0.0030	0.0202 ± 0.0030	0.0390 ± 0.0030	0.0347 ± 0.0083
SRX mantATPase contribution (1/T_SRX_)*P_SRX_	0.0018 ± 0.0002	0.0025 ± 0.0003	0.0020 ± 0.0006	0.0025 ± 0.0092
Chasing estimated mantATP rate (1/s)	0.0290 ± 0.0033	0.0228 ± 0.0033	0.0410 ± 0.0126	0.0373 ± 0.0089
Measured mantATPase rate (1/s)	0.0208 ± 0.006		0.0379 ± 0.0083	
Error (%)	+28.5	+8.8	+7.6	−1.6

Populations and time constant parameters obtained from 3-exp fitting (method 2) and 3-exp fitting considering the NSP (method 3) are reported on the first 4 rows. The table shows the calculations performed to estimate mantATP consumption based on chasing parameters, the values are then compared with the ones obtained by NADH coupled reactions ATPase assay reported in Fig. 6*B*. In the last raw the error differences between estimated and measured ATPase rate for each condition (piperine, DMSO) and each analysis model are reported.

## Discussion

In this work, we evaluated the NSPs contributing to the fluorescent signal decay in a muscle fiber chasing experiment. We applied a ghosted fibers model in combination with para-aminoblebbistatin to exclude myosin from skinned fibers and characterize the nonspecific signal amplitude and kinetics. The study was also enriched with a proteomic analysis which validated myosin extraction, possibly suggesting the contribution of other structural proteins and ATPases to the nonspecific mantATP signal.

Our findings using ghosted fibers suggest that the NSP constitutes 60% of the initial fluorescent emission. We demonstrated that the kinetics of this component partially overlaps with that of the disordered myosin population, possibly leading to an overestimation of the fast-cycling myosin population. We quantitatively estimated its influence on three different exponential fitting techniques. The double exponential fit did not adequately describe the populations, since the DRX component is strongly overestimated.

The standard three exponential fitting performed generally better than the double exponential. However, the higher is the SRX population, the lower is the reliability of this method. In this scenario, the data obtained by three exponential fitting need to be interpreted considering the nonspecific signal. As already reported in the literature, the triple fitting method can only be applied if experimental sampling rate is adequately high (26). Finally, we measured the hydrolysis rate of mantATP in control fibers, and we compared it with the estimated mantATP turnover rate obtained from populations and time constants of the chasing assay. The agreement of these two values suggests the reliability of our approach. In the presence of piperine, the increased fast cycling population caused the direct DRX estimation from the three exponentials fitting to give more reliable parameters, despite the NSP corrected populations still leading to the best estimation.

Our Monte-Carlo model based on the most recent information on inter- and intraproteins cooperativity (15, 27), reproduced the multiexponential decay even when the motors were individually in thermodynamic equilibrium. The multiexponential decay can be observed when at least two sets of motors with different SRX and DRX turnovers are considered, a configuration possibly not available in a purified myosin S1 preparation. However, our simulations indicate that the intradimer cooperativity, where the rates of the blocked head are affected by the state of the free head in the same dimer, can be enough to generate a multiple decay signal. In this context, a similar decay could be obtained also from two-headed heavy meromyosin (HMM) preparations. However, this intradimer cooperativity may be due to additional constraints peculiar to intact filaments, such as interactions with the light meromyosin, MyBP-C or titin, that are lost in the HMM protein construct. In those cases, a single exponential would appear, as demonstrated by Moran et al. (10). We hypothesized that native thick filaments maintain a fluid equilibrium of myosin heads distributed across a continuum of biochemical states, influenced by structural features and cooperative interactions. Despite this complexity, the coherence of the chasing parameters with the ATPase hydrolysis rate strongly suggests that the simplification of considering only two independent populations still preserves enough details to describe the system.

It has been inferred that in a physiological context, the resting thick filament is characterized by a large predominance of myosins in SRX (1). Skinned fiber preparations have shown thick filament structural alterations (28, 29) that could affect the stability of biochemical states. It is possible to assume that the skinning procedure would enrich the disordered heads population rather than causing an increased SRX, widening the gap between an in vivo configuration and the experimental setting. In the interest of clinical interpretation of myosin resting states, an improved skinned fiber preparation may be required (e.g., addition of dextran to restore lattice spacing and myosin orientation). It is known that mantATP is susceptible to photobleaching, and that the minimization of this effect is needed to avoid slow component quantification errors (10). The setup used to perform the experiments reported in this work has been optimized to avoid mantATP signal loss due to photobleaching (optimal excitation and emission wavelengths, ultraviolet radiation (UV) suitable lenses, very sensitive camera). We chose to leave this topic out of the manuscript because we considered it relatively straightforward to evaluate and mitigate. Nonspecific signal amplitude and photobleaching strongly depend on the preparation quality and on the experimental setup, our suggestion is to accompany the experimental optimization with an adequate analytical approach as the one reported here. In this context, the use of ghost fibers as a control system to determine NSPs can help reduce variability among different skinned fiber preparations. In this work, we confirmed what was suggested previously (10), that the convergence of ATPase rate and mantATP chasing parameters is a strong indicator of the validity of experimental procedures and analysis.

Developing the mantATP chasing experiment, Cooke et al. identified two biochemical states in resting muscle fibers, opening a new field in the study of fundamental myology. With this work, we aim to incorporate this important advancement by providing guidance on properly conducting mantATP chasing experiments and reliably analyzing their results.

## Materials and Methods

### Modeling.

For the numerical simulations we used two approaches: a Monte-Carlo model of a single half-sarcomere to simulate the role of myosin motors cooperativity in the nucleotide hydrolyzation rate, and a reaction–diffusion model to simulate the decay of mantATP concentration into a whole fiber, due to its hydrolysis and diffusion into an external bath.

### Monte-Carlo Model.

We modified our previously published sarcomere model (30) to include the biochemical states of SRX and DRX. A built-in pseudorandom number generator was used to simulate the likelihood of both the SRX-DRX transition and the mantATP hydrolysis in every head at each time step Δt = 10 µs.

In all models (except for model 0a in the *SI Appendix*), heads are oscillating between SRX and DRX state, DRX heads (green in Fig. 1*B*), have a mantATP hydrolyzation rate of 0.05 s^−1^, while SRX heads (purple in Fig. 1*B*) hydrolyze mantATP at a rate of 0.005 s^−1^. Models 0, and 1 (see text and Fig. 1) simulates 147 myosin dimers, assuming the same behavior for both heads. In Model 2 and 3, the two heads in each dimer were treated independently, to allow intra- and interdimer cooperativity. In each filament, the motors are indexed from 1 to 294, being the even number associated to the blocked heads and the odd number to the free heads. A blocked head in the SRX state cannot switch to DRX if its free head is still in its SRX state. In the same way, a free head in the DRX state cannot switch to SRX if its blocked head is in its DRX state. In model 3, free heads in each dimer have a lower *k*_SRX-DRX_ rate and a higher *k*_DRX-SRX_ rate, if the previous dimer toward the M-line has one or two heads in the SRX state (intramolecular cooperativity). The sarcomere model is available at https://github.com/lorenzomarcucci/mantATPase.

### Reaction–Diffusion Model.

We used the geometrical scheme represented in Fig. 1*D*, with a rotationally symmetric fiber divided into nine concentric cylinders, each progressively incremented by 4 µm in radius, resulting in a 72 µm fiber in diameter. Free and bound mantATP is initially in equilibrium inside the fiber to reach the experimental value of 250 µM of free mantATP. Unlabeled ATP is present in the compartments outside the fiber at a constant concentration of 4 mM (Fig. 1*D*). The diffusion of the two nucleotides is then modeled as in ref. 31, with a diffusion coefficient inside the solution of 6 × 10^2^ µm^2^ s^−1^ (32), and inside the fiber of one fifth of this value, as obtained from the ghosted fibers experimental data. The two nucleotides are competitors for binding the myosin motors and binding and unbinding rates for ATP are assumed to be one order of magnitude greater than mantATP (25). mantATP can also bind to nonspecific sites uniformly distributed inside the fiber. Reactions are described through the general equation for the specific and nonspecific binding:

$$\frac{d\left[SmATP\right]}{\mathrm{dt}}=k_{\mathrm{ON}}{\left[mATP\right]}^{n}\left[S\right]-k_{\mathrm{OFF}}\left[SmATP\right],$$

where [*S*] is the free buffer concentration, [*SmATP*] the bound buffer concentration, with [*Stot*] = [*SmATP*] + [*S*] and n simulates the cooperativity of the reaction, equal to 1 if not present. The nucleotides diffuse and react, and the total amount of mantATP inside the fiber is assumed to be the fluorescence observed experimentally. The myosin concentration has been imposed to 105 µM, based on the 180 µM (33, 34), and adjusted for the swelling of skinned muscle fibers that is estimated to be 125% (35, 36). The fiber model is available at https://github.com/lorenzomarcucci/Mantdiffusion.

### Fitting Techniques.

We explored three possible ways, proposed in the literature, to interpret SRX and DRX populations from the mantATP chasing data.

1) Two exponential fitting of the normalized fluorescence decay *F*(*t*)/*F*_0_ though an equation of the type:

$$F(t)/F_{0}=1-P_{\text{SRX}}\left(1-e^{-t/T_{\text{SRX}}}\right)-P_{\text{DRX}}\left(1-e^{-t/T_{\text{DRX}}}\right),$$

2) Three exponential fitting equation of the type

$$F(t)/F_{0}=1-P_{\text{NSP}}\left(1-e^{-t/T_{\text{NSP}}}\right)-P_{\text{SRX}}\left(1-e^{-t/T_{\text{SRX}}}\right)-P_{\text{DRX}}\left(1-e^{-t/T_{\text{DRX}}}\right),$$

where the relative populations are obtained from SRX = P_SRX_/(P_DRX_+P_SRX_) and DRX = 1 − SRX.

3) Three exponential fitting equation as in 2, where the SRX absolute population is given by P_SRX_, but DRX is deduced from an independent estimation of the NSP from the relation DRX = 1 − NSP − P_SRX_ so that:

$$\text{DRX}=\left(1-\text{NSP}-P_{\text{SRX}}\right)/\left(1-\text{NSP}\right)=1-P_{\text{SRX}}/\left(1-\text{NSP}\right).$$

A similar approach has been used in ref. 1.

### Muscle Samples and Solutions.

No living animals were involved in the current study as all measurements have been performed on ex vivo tissues. Animal handling and killing were performed by trained personnel following the local animal welfare guidelines. White adult New Zealand rabbits were killed according to protocols approved to Prof. Oriano Marin (Dept. Biomedical Sciences, University of Padova) who kindly agreed to share muscle tissue. Bundles of the psoas muscle were harvested and skinned as described in ref. 37. To set up each of the methods described below, a thin bundle of fibers was dissected and incubated for 10 min in a relax buffer (120 mM potassium acetate, 50 mM 3-(N-morpholino)propanesulfonic acid (MOPS), 9 mM ethylenediaminetetraacetic acid (EGTA), 6 mM MgCl_2_, 6 mM KH_2_PO_4_, 4 mM ATP, 1 mM dithiothreitol (DTT), pH 7.0) containing 0.5% Triton X-100 at 4 °C, then washed three times using a fresh relaxing buffer with 0.025% Triton-X100.

### Ghosted Fibers.

Individual permeabilized fibers were dissected and mounted on aluminum T-clips in chasing buffer (potassium acetate 120 mM, MOPS 50 mM, EGTA 4 mM, KH_2_PO_4_ 5 mM, magnesium acetate 5 mM, DTT 1 mM, 4 mM ATP pH 6.8) between a 403C force transducer and a 315D motor of an Aurora Scientific 802D single fiber system. The bath temperature is set to 10 °C and myosin is extracted with a relax buffer supplemented with 0.5 M KCL and 0.01 M Na_4_P_2_O_7_ for 25 min (17, 18, 38). After myosin extraction, the isometric tension developed in a calcium saturated solution was nearly zero. Myosin extraction extent was evaluated by densitometry of electrophoresis gel analysis combined with silver staining coloration (*SI Appendix*, Fig. S2 *A* and *B*).

### Confocal Analysis.

Permeabilized single fibers were mechanically isolated and mounted on an aluminum clip previously glued to a 25 mm coverslip (cat. 01.4150.31, Vetrotecnica, Italy) that was mounted into an Attofluor cell chamber (A7816, Invitrogen). Fibers were incubated in relax chasing buffer with 1:750 dilution of Alexa Fluor 568 phalloidin (A1238, lot. 2077757, Life Technologies) for 10 min at room temperature. Phalloidin-relax buffer was then changed with three 5-min washes of rigor buffer (relax buffer without ATP). Before leading to images acquisition the fibers were incubated for 5 min in relax buffer containing 250 µM mantATP (NU-202L, Jena Bioscience, Germany) instead of regular ATP. For ghost fibers visualization, the fibers were incubated in an extraction buffer (relax ATP buffer with 0.5 M KCl and 0.01 M Na_4_P_2_O_7_) for 25 min at 4 °C. Images were acquired using a Zeiss LSM900 inverted confocal microscope at 63× magnification. Phalloidin and mantATP fluorescence were excited with solid state lasers at 561 nm and 405 nm and acquired at 567 to 700 nm and 400 to 500 nm respectively. All the images were acquired at 1 A.U. and analyzed using Fiji (39). For each fiber a 10 to 15 sarcomeres length region-of-interest was analyzed and the fluorescence signal was normalized on the respective background outside the fiber.

### Mass Spec Analysis.

Ghost fibers (n = 7) and myosin extracted fraction (n = 7) were lysed with 1% sodium dodecyl sulfate in phosphate-buffered saline, 15 mM 2-Chloroacetamide, and 5 mM tris(2-carboxyethyl)phosphine, heated to 95 °C for 10 min, and sonicated using the bioruptor (Bioruptor Pico, Diagenode, Seraing, Belgium) with 10 cycles of 30 s and 30 s break. Protein digestion was performed following the SP3 protocol (40). In brief, both hydrophilic and hydrophobic beads were added to the sample and bound by adding 1:1 volume of acetonitrile (ACN). After 8-min incubation time, magnetic beads were immobilized and washed 2× with 70% ethanol and ACN. Proteins were digested with trypsin (substrate:enzyme ratio 100:1) overnight at room temperature. Samples were acidified by using 100 µL 0.1% formic acid (FA) followed by a clean-up with house-made SDB-reverse phase sulfonate tips. Desalted peptides were loaded on EvoTips Pure. Peptide separation was performed on an EvoSep One system (both Evosep, Denmark) equipped with a 5 cm IonOptics column, 75 µm inner diameter (IonOpticks, Australia) with the preprogrammed 80 SPD whisper method. Mobile phases consisted of 0.1% FA as solvent A and 0.1% FA in ACN as solvent B. The HPLC system was coupled to an Orbitrap Astral using Nanospray Flex ion source (both Thermo Fisher Scientific). The Orbitrap Astral was operated with a resolution setting of 240,000 for full MS scans across a mass-to-charge range of 380 to 980 *m/z*. The automatic gain control for full MS was adjusted to 500%. Fragment ion scans were performed at a resolution of 80,000 with a maximum injection time of 6 ms, with 300 windows implemented of four thomson unit (mass-to-charge ratio) (TH) spanning the 380 to 980 *m/z* range. The isolated ions underwent fragmentation using Higher-energy Collisional Dissociation with a normalized collision energy of 25%.

The mass spectrometry proteomics data have been deposited to the ProteomeXchange Consortium via the PRIDE partner repository with the dataset identifier PXD060451 (41). Acquired spectra were analyzed with DIA-NN (V1.9.2) using library free search against UniProt Oryctolagus cuniculus (Rabbit) database (January, 2025) (42). Mass ranges were set according to the settings of the mass spectrometer. Data were further processed using R (V 4.2.2), with the libraries: tidyverse, diann, data.table, magrittr, FactoMineR, factoextra and ggplot2, gprofiler, ggplot2. Data input was filtered for unique peptides, q-Value <0.01, Lib.Q.Value<0.01, PG.Q.Value<0.01, Global.Q.Value<0.01, Quantity.Quality>0.7, Fragment.count>=4. Protein intensities were normalized to the sum intensities of the matching extracted and fiber proteomes (“fiber” and “MYH extraction”). Statistical analysis was performed using ordinary two-way ANOVA, Sidak’s multiple comparisons test single pooled variance, data are reported as mean ± SEM.

### mantATP Chasing.

Single fibers were dissected from rabbit psoas bundles and mounted on aluminum T-clips and into an Aurora scientific 802D setup in chasing buffer (1). One end was mounted on the tensiometer hook, while the other was mounted on the motor hook to control fiber length. The sarcomere length was adjusted to 2.4 to 2.5 µm. The fiber was mounted in relax buffer and then incubated in a rigor buffer (potassium acetate 120 mM, MOPS 50 mM, EGTA 4 mM, KH_2_PO_4_ 5 mM, magnesium chloride 5 mM, DTT 1 mM, pH 6.8) for 5 min at 10 °C. After the incubation, the fiber was moved to a well containing 250 μM mantATP (NU-202L, Jena Bioscience, Germany) relax buffer and incubated for 5 min. At the beginning of the recording, fiber was moved to a new well containing a fresh relax buffer with 4 mM ATP. Images were acquired every 2 s for a total measurement time of 800 s and the acquisition time for every frame was 300 ms (while sample illumination was approximately 0.4 s per cycle). The decrease in fluorescence intensity was fitted with the functions explained in the section “Fitting techniques.”

All measurements were carried out at controlled temperature of 24 °C and the acquisition were performed by using the single fiber setup placed on an Olympus IX70 inverted fluorescent microscope, equipped with a 20× objective (EVIDENT LUCPLFLN 20×/0.45), a THORLAB 365 nm light-emitting diode (LED) (M365LP1) controlled by a LEDD1B driver used as a light source and a Hamamatsu ORCA Flash-4.0 camera (C13440-20CU) to capture the emission. A self-compiled Arduino system was used to synchronize triggers of LED activation, camera acquisition, and Aurora baths movement.

### NADH Coupled Reaction ATPase Activity Assay.

Single fibers were dissected and pipetted into a 384-well plate in ATPase buffer (K_2_CO_3_ 20 mM, MOPS 60 mM, EGTA 5 mM, MgCl_2_ 7.2 mM, KH_2_PO_4_ 4.8 mM, ATP 6 mM, DTT 1 mM, Triton X-100 0.025%, pH 7.3) plus the coupled reaction buffer (NADH 1 mM, PEP 5 mM, pyruvate kinase P1506-25KU Sigma Aldrich 40 U/mL and Lactate Dehydrogenase L2625-25KU Sigma Aldrich 40 U/mL) to a final volume of 30 μL. ATPase analysis using mantATP was carried out using 400 U/mL of Pyruvate Kinase 80 U/mL of Lactate Dehydrogenase, and with the buffer containing 400 µM mantATP instead of normal ATP (ionic strength adjusted to about 180 mM). In experiments including the SERCA inhibitor, a final concentration of 0.1 µM thapsigargin (cat. S7895, Selleckchem) was included in the buffer, while to inhibit myosin para-aminoblebbistatin (CAS. 2097734-03-5, Cayman Chemical) was used. The plate was covered with an optically clear seal (cat. 95.1994, Sarstedt) and placed in a temperature controlled multiplate reader set to 27 °C (Multiskan SkyHigh, ThermoFisher Scientific). The coupled reaction acts as described in refs. 43 and 44. The oxidation rate of NADH was measured every 2 min as the decreasing absorbance at 340 nm, total run time 25 min. The linear decrease was estimated using NADH absorbance epsilon of 6,300 mol^−1^ cm^−1^ and using a calibration curve obtained by the addition of a growing concentration of ADP and mantADP. A concentrated KCl solution was added to each well at the end of the assay to reach the final concentration of 0.4 M and extract myosin from fiber, the protein amount was measured using Pierce™ 660 nm Protein Assay Reagent (Thermo Fisher nr. 22660) for normalization purposes.

### Statistical Analysis.

Statistical analysis was performed using GraphPad Prism (version 10.0.2 for Mac, GraphPad Software, Boston, MA). Except when stated differently, statistical analysis was performed validating normal distribution, then applying one-way ANOVA or *t* test analysis in which significance is reached upon *P* < 0.05. In Fig. 4*A*, normal distribution was assessed using a Shapiro–Wilk test, and unpaired *t* test was used among ctrl vs. ctr_Ablebb, and individual ghosted vs. controls comparisons. In Fig. 6*B*, outliers were identified using the ROUT method (Q = 0.5%), then normality distribution was assessed by the Kolmogorov–Smirnov test, and an ordinary one-way ANOVA with Tukey’s multiple comparisons test was used to highlight significance (*P* < 0.05).

## Supplementary Material

Appendix 01 (PDF)

## Data Availability

Proteomic data have been deposited in ProteomeXchange Consortium with the dataset identifier (PXD060451) (45). All the data generated in this study are provided within the manuscript and the *SI Appendix*.
