# Supplementary material for: A combined experimental and computational analysis of mantATP turnover in skinned muscle fibers
Source: Proc Natl Acad Sci U S A. 2025 May 15;122(20):e2502652122. doi: 10.1073/pnas.2502652122 (PMC12107101; doi:10.1073/pnas.2502652122)
Supplement: Supplementary file 1 — Appendix 01 (PDF) [file pnas.2502652122.sapp.pdf]

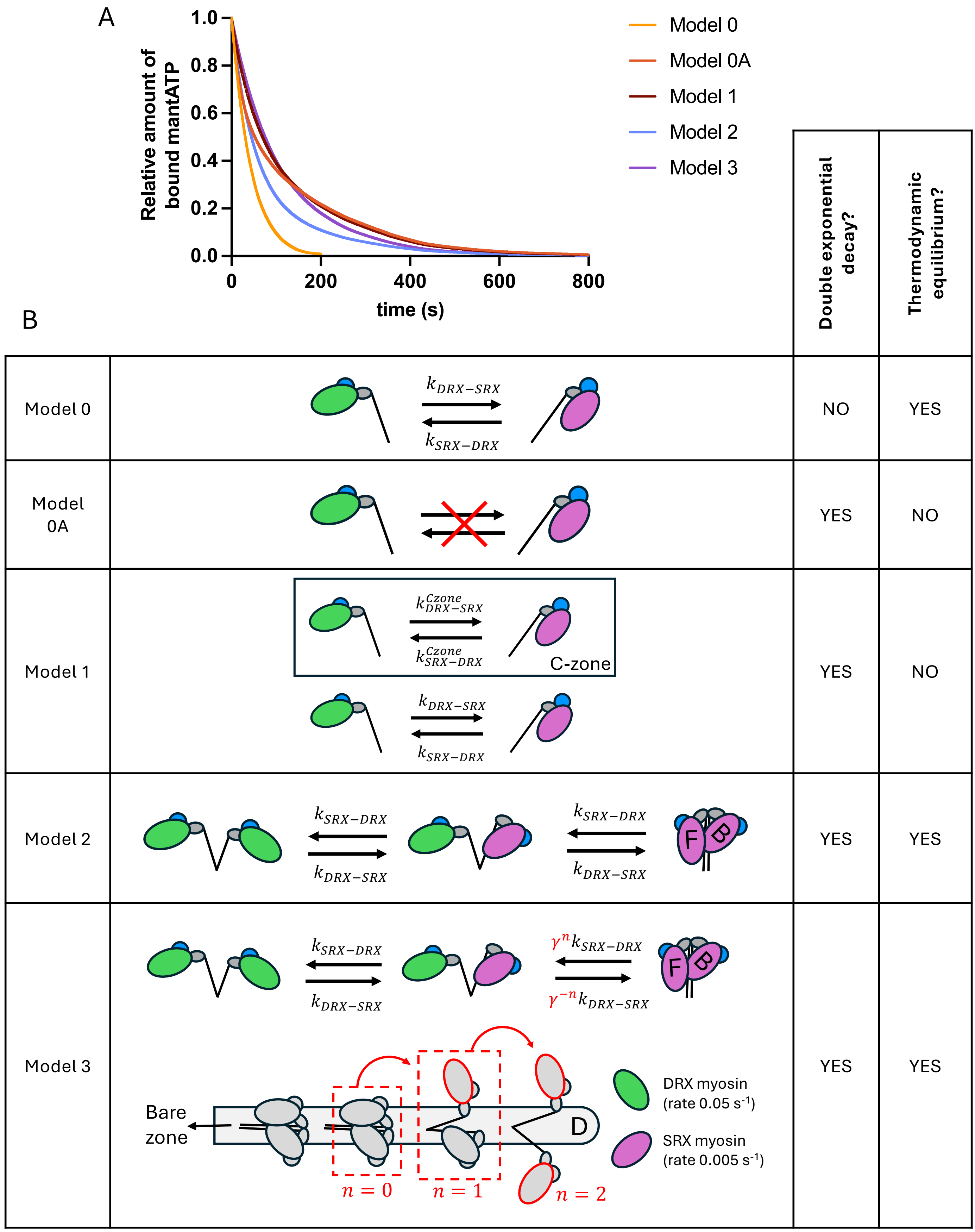

Fig. S1. (A) Simulated amount of bounded mantATP in a single sarcomere under different hypotheses for the cooperativity between myosin motors. Model 0: non cooperative thermodynamically equilibrated motors. Model 0A: non cooperative motors not in thermodynamical equilibrium. Model 1: two populations of motors defined by a structural constrain (e.g. myosin binding protein C). Model 2: two populations of motors with diverse thermodynamic equilibrium due to intradimer cooperativity. Model 3: fully cooperative model with both intradimer and intermolecular cooperativity. **(B)** Schematic representations of the models described in panel A.

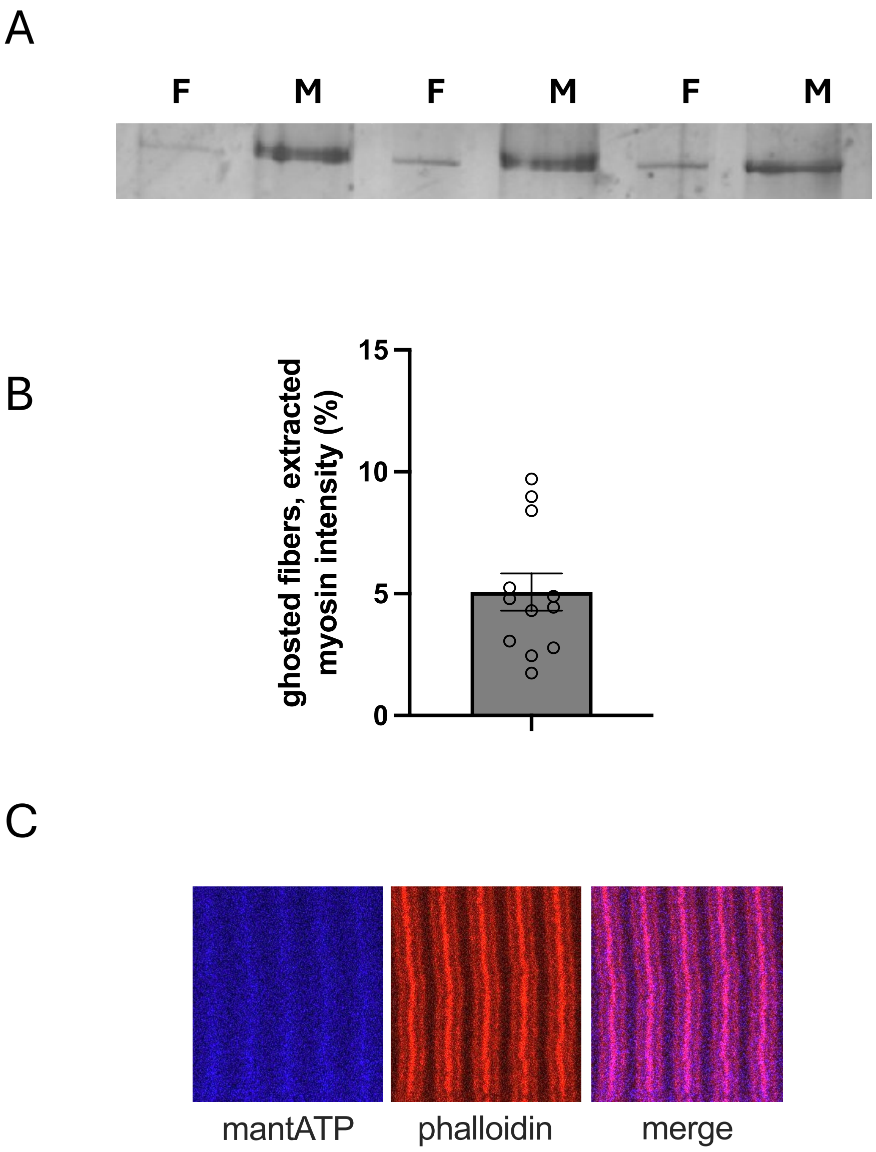

Fig. S2. **(A)** silver staining of skinned fibers with myosin extracted (lines marked with M) and the remaining amount within fibers (lines marked with F). Myosin left is estimated to be around 5.06+/-0.75% (n=12, mean+/-SEM). **(B)** Estimation of the amount of myosin left in the skinned muscle fiber after the ghosting protocol. Values are obtained by densitometry analysis of silver-stained SDS-page gel electrophoresis of extract compared to matching fiber (n=12, mean+/-SEM). **(C)** In ghosted fibers mantATP signal (blue) and phalloidin signal (red) colocalize in the merged picture (magenta). High magnification confocal image of 5 sarcomeres taken at the confocal microscope.

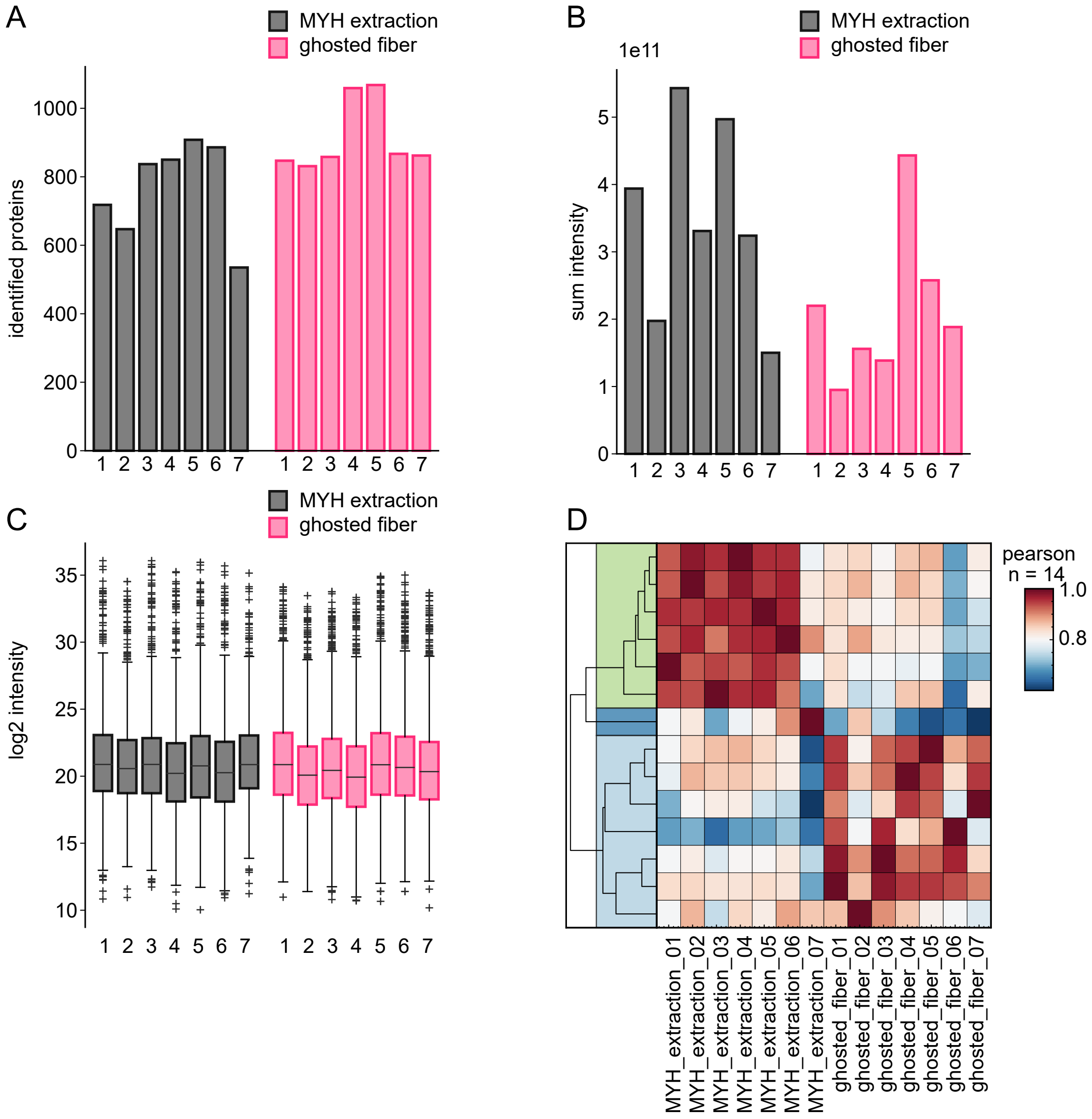

Fig. S3. (A) number of identified proteins in each sample, samples are originated from 7 single fibers so that extraction samples (black) are matching the corresponding ghosted fiber (magenta). **(B) and (C)** sum of intensities in extraction samples (black) and in ghosted fiber samples (magenta), log2 of individual intensities reveals a similar variability among peptides. **(D)** Heat map showing hierarchical clustering of median normalized Z-scores for mixed fibers in extracted proteins and proteins left in the ghosted fiber.

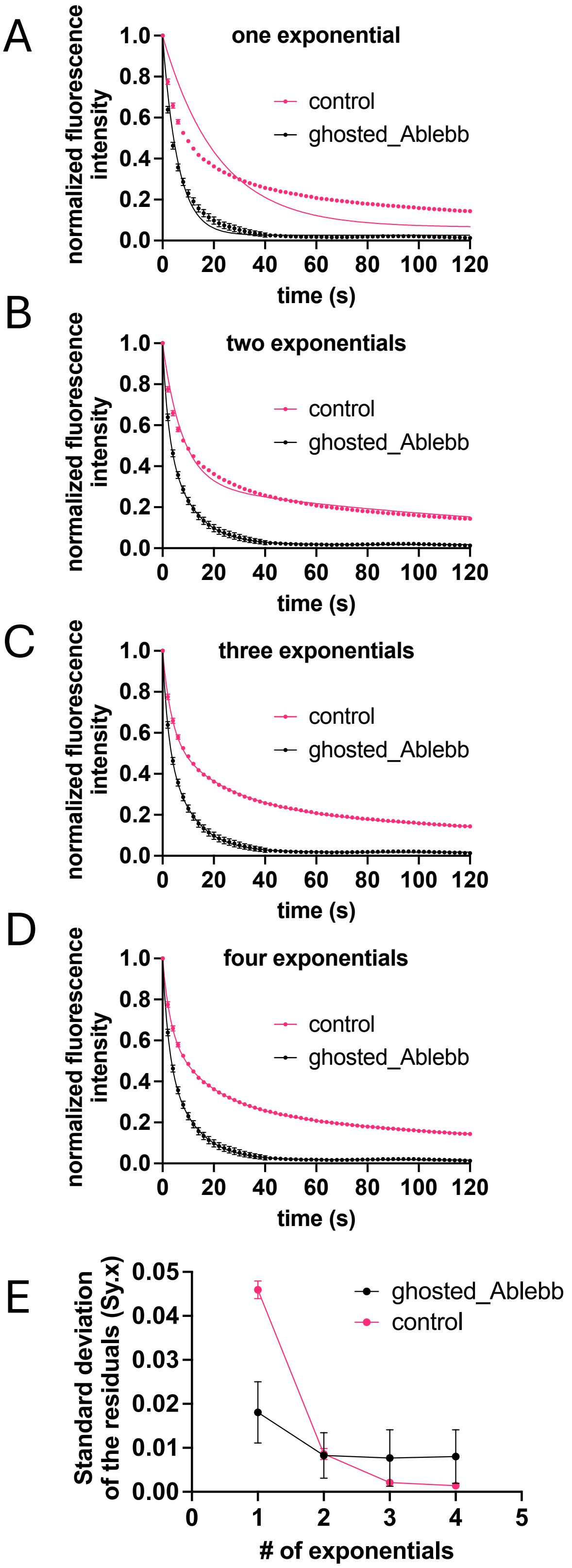

single_exp_decay		
Best-fit values	ghosted_Ablebb	control
P1	0.987	0.935
T1	6.337	21.543
Goodness of Fit		
R squared	0.970	0.800
Sum of Squares	0.057	0.841
Sy.x	0.018	0.046
double_exp_decay		
Best-fit values	ghosted_Ablebb	control
P1	0.462	0.683
T1	2.222	7.708
P2	0.529	0.285
T2	14.212	140.663
Goodness of Fit		
R squared	0.993	0.993
Sum of Squares	0.014	0.030
Sy.x	0.008	0.009
triple_exp_decay		
Best-fit values	ghosted_Ablebb	control
P0	0.418	0.413
T0	2.023	3.554
P1	0.558	0.353
T1	11.016	23.806
P2	0.021	0.212
T2	123.290	207.825
Goodness of Fit		
R squared	0.993	1.000
Sum of Squares	0.014	0.002
Sy.x	0.008	0.002
four_exp_decay		
Best-fit values	ghosted_Ablebb	control
P0	0.388	0.290
T0	2.166	5.232
P1	32.115	0.356
T1	10.695	12.921
P2	3.272	0.231
T2	663.097	110.143
P3	-16.005	0.168
T3	1475.110	559.817
Goodness of Fit		
R squared	0.993	0.544
Sum of Squares	0.014	1.766
Sy.x	0.008	0.025

Fig. S4. Fitting of chasing of control and ghosted para-aminoblebbistatin fibers with an increasing number of exponentials. (A) single exponential, **(B)** two exponentials, **(C)** three exponentials and **(D)** four exponentials. The table on the right summarizes the parameters obtained for the corresponding fitting, in red those reported in Figure 4. **(E)** Decrease of standard deviation of the residuals of the fitting reported above. For ghosted fibers in para-aminoblebbistatin (black), after the second exponential there is no decrease in Sy.x, meaning that the additional term does not improve the fitting. For control fibers (magenta), the third exponential does improve the fitting, while the fourth one did not.

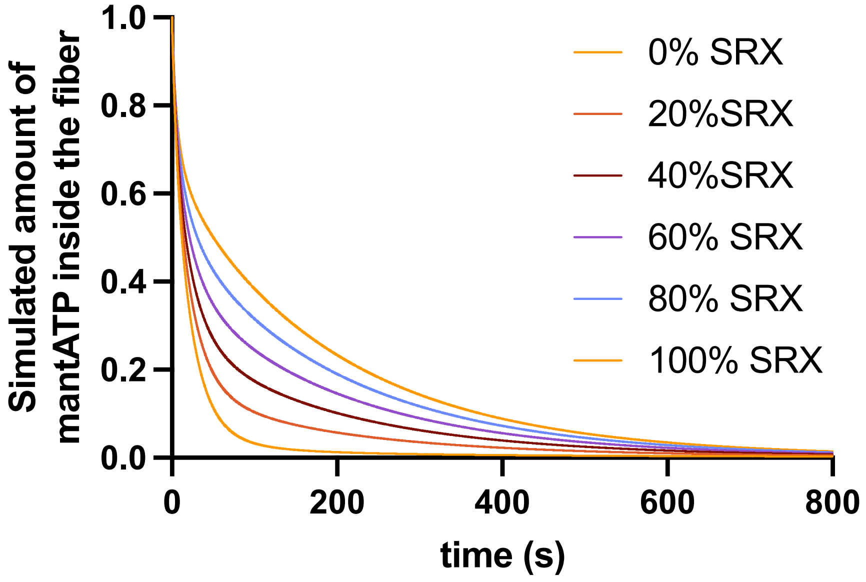

Fig. S5. Simulated decays at an imposed NSP=60% and various SRX-DRX populations. The diffusional model is used to simulate the time variations of the mantATP concentration inside the fiber when the nonspecific component account for the 60% of the total concentration at time t=0 and at various relative amounts of SRX and DRX motors, from 0% to 100% with steps of 5% (only 20% steps are reported in the graph). The decay has a qualitatively similar behaviour as the experimental ones present in literature at various SRX populations, with an increase of the signal at the same time, at the increase of the SRX imposed population.

Model	P_{DRX} (%)	P_{SRX} (%)	T_{DRX} (s)	T_{SRX} (s)
0	100.0	-	44.0	-
0A	40.0	60.0	20.0	191.0
1	33.0	67.0	40.5	175.0
2	66.0	34.0	43.6	168.7
3	36.0	64.0	67.6	145.1

Table S1. One- or Two-exponential fitting parameters for the models described in the text. When all myosin motors behave in the same way (Model 0), like in purified proteins, a single exponential can fit the predicted decay of the population of myosin bound to a mantATP. For models description see main text.

P_{SRX} and P_{DRX} imposed to 50%				
**Imposed nonspecific (%)**	**2-exponential fitting**			
	**estimated P_{SRX} (%)**	**estimated P_{DRX} (%)**	**estimated T_{SRX} (s)**	**estimated T_{DRX} (s)**
20	46.5	52.5	166.1	10.2
40	38.8	60.0	146.6	7.0
60	30.0	68.8	129.6	6.4
80	24.8	74.0	97.9	7.7
**Imposed nonspecific (%)**	**3-exponential fitting (P_{DRX} = 1- P_{SRX})**			
	**estimated P_{SRX} (%)**	**estimated P_{DRX} (%)**	**estimated T_{SRX} (s)**	**estimated T_{DRX} (s)**
20	49.3	50.7	198.9	19.8
40	45.3	54.7	195.7	18.9
60	37.4	62.6	188.0	17.4
80	23.7	76.3	163.2	16.3
**Imposed nonspecific (%)**	**3-exponential fitting (P_{DRX} = 1 - P_{ASP} - P_{SRX})**			
	**estimated P_{SRX} (%)**	**estimated P_{DRX} (%)**	**estimated T_{SRX} (s)**	**estimated T_{DRX} (s)**
20	51.4	48.6	198.9	19.8
40	53.2	46.8	195.7	18.9
60	57.7	42.3	188.0	17.4
80	80.2	19.8	163.2	16.3

Table S2. (A) Estimated parameters from three different fitting techniques applied to the simulated decay of mantATP concentration inside a muscle fiber with imposed 50% of relative SRX (and DRX) and variable imposed nonspecific components. Imposed time constants are T_{SRX}=200 s^{-1} and T_{DRX}=20 s^{-1}. Parameters obtained with the two exponential, three exponential and three exponential plus independent estimation of the NSP are reported in the upper, middle and lower panel, respectively. The three-exponential fitting always reaches a better prediction than the two exponential fitting methods. The three methods have similar performances at low NSP. Two-exponential approach failed to keep a low error already at NSP=40%, the three-exponential approach with the use of P_{DRX} has an error higher than 10% at NSP=60%, the value obtained experimentally in this work. The three-exponential approach with the independent estimation of NSP kept the error low even at NSP=60%. At 80% this approach overestimates the imposed SRX (16% estimated vs. 10% imposed in absolute terms)and induces a relatively high underestimation of the DRX (100-80-16=4%, vs 10% in absolute terms). In relative terms this results in an 80%-20% estimation.
